# LncRNAs as biomarkers for predicting radioresistance and survival in cancer: a meta-analysis

**DOI:** 10.1038/s41598-022-21785-1

**Published:** 2022-11-02

**Authors:** Yuxin Xie, Jiaqi Han, Keqi Xie, Qiheng Gou

**Affiliations:** 1 Breast Disease Center, Cancer Center, West China Hospital, Sichuan University, 37 Guoxue Xiang, Wuhou District, Chengdu, 610041 China; 2 Laboratory of Molecular Diagnosis of Cancer, Clinical Research Center for Breast, West China Hospital, Sichuan University, Chengdu, Sichuan China; 3 Departments of Anesthesiology, Mianyang Central Hospital, Mianyang, Sichuan China; 4 Department of Radiation Oncology and Department of Head & Neck Oncology, Cancer Center, West China Hospital, Sichuan University, Chengdu, China

**Keywords:** Cancer, Cell biology, Biomarkers

## Abstract

The effect of long noncoding RNAs (lncRNAs) on the radiotherapy response has been gradually revealed. This systematic review and meta-analysis aimed to evaluate the association between the function and underlying mechanism of lncRNAs in regulating the radiosensitivity and radioresistance of different tumors. Hazard ratios (HRs) with corresponding 95% confidence intervals (CIs) were calculated to estimate the effect of lncRNAs on cancer patient prognosis, including overall survival (OS), recurrence-free survival (RFS), disease-free survival (DFS) and progression-free survival (PFS). Collectively, 23 lncRNAs in 11 cancer types were enrolled. Of them, 13 lncRNAs were downregulated and related to radiosensitivity, 11 lncRNAs were upregulated and related to radioresistance, and 3 lncRNAs were upregulated and related to radiosensitivity in cancers. Furthermore, 17 microRNAs and 20 pathways were targeted by different lncRNAs and contributed to the cancer radiotherapy response in this meta-analysis. The individual pooled HRs (95% CIs) of downregulated radiation-resistant and upregulated radiation-resistant lncRNAs for OS were 0.49 (0.40–0.60) and 1.88 (1.26–2.79), respectively. Our results showed that lncRNAs could modulate tumor radioresistance or sensitivity by affecting radiation-related signaling pathways and serve as potential biomarkers to predict radiotherapy response.

## Introduction

Multiple treatment options, including surgery, chemotherapy, radiotherapy, targeted therapy, and immunotherapy, have been applied to improve the survival of cancer patients. Of them, radiotherapy is carried out in the treatment of more than half of malignancies^1^. Radiation contributes to the formation of reactive oxygen species (ROS) and free radicals that lead to DNA single-strand breaks (SSBs) and double-strand breaks (DSBs), ultimately causing cancer cell death^2^. However, radiotherapy resistance limits its efficacy and has become a major clinical challenge. Although the mechanisms of radioresistance in cancer cells are not clear, they likely involve a series of biological and genetic alterations, such as aberrant DNA damage response (DDR) and enhanced DNA damage repair, disorder of cell proliferation and apoptosis, and related abnormal activation molecular mechanisms^3,4^.

Long noncoding RNAs (lncRNAs) are defined as transcripts of more than 200 nucleotides (nt), and the vast majority of them are not translated into proteins^5,6^. Recently, accumulating evidence has investigated the role of lncRNAs in regulating chromatin remodeling, transcription and cell biological behavior, such as proliferation, migration, immortality, and angiogenesis, in malignancies^7,8^. In particular, the relationship between lncRNAs and the cancer response to radiotherapy has received increased attention. Studies have shown that lncRNAs can modulate radioresistance through multiple processes, such as DNA damage repair, apoptosis, epithelial-mesenchymal transition (EMT) and cancer stem cell (CSC) activity^3,9,10^. For instance, the lncRNA DNM3OS has been confirmed to inhibit irradiation-induced DDR and confer significant radioresistance in esophageal squamous cell carcinoma (ESCC)^11^. LncRNA LINC02582 could promote breast cancer radioresistance by interacting with deubiquitinating enzyme ubiquitin specific peptidase 7 (USP7) to deubiquitinate and stabilize checkpoint kinase 1 (CHK1), a key molecule in DDR^12^. Inhibition of lncRNA HOTAIR could facilitate apoptosis, inhibit autophagy, and enhance the radiosensitivity of CRC after irradiation by regulating the miR-93/ATG12 axis^13^. LncRNA RBM5-AS1 was proven to be a novel inducer of medulloblastoma stemness, and silencing of RBM5-AS1 enhanced irradiation-induced apoptosis and DDR in medulloblastoma cells^14^.

These findings imply the potential use of lncRNAs as predictors or diagnostic biomarkers for identifying radioresistant patients in the clinic. Thus, in this study, we performed a systematic review and meta-analysis to investigate and classify the association of lncRNAs with cell response to radiotherapy and prognosis in different types of carcinomas.

## Materials and methods

### Search strategy

**Figure 1:**
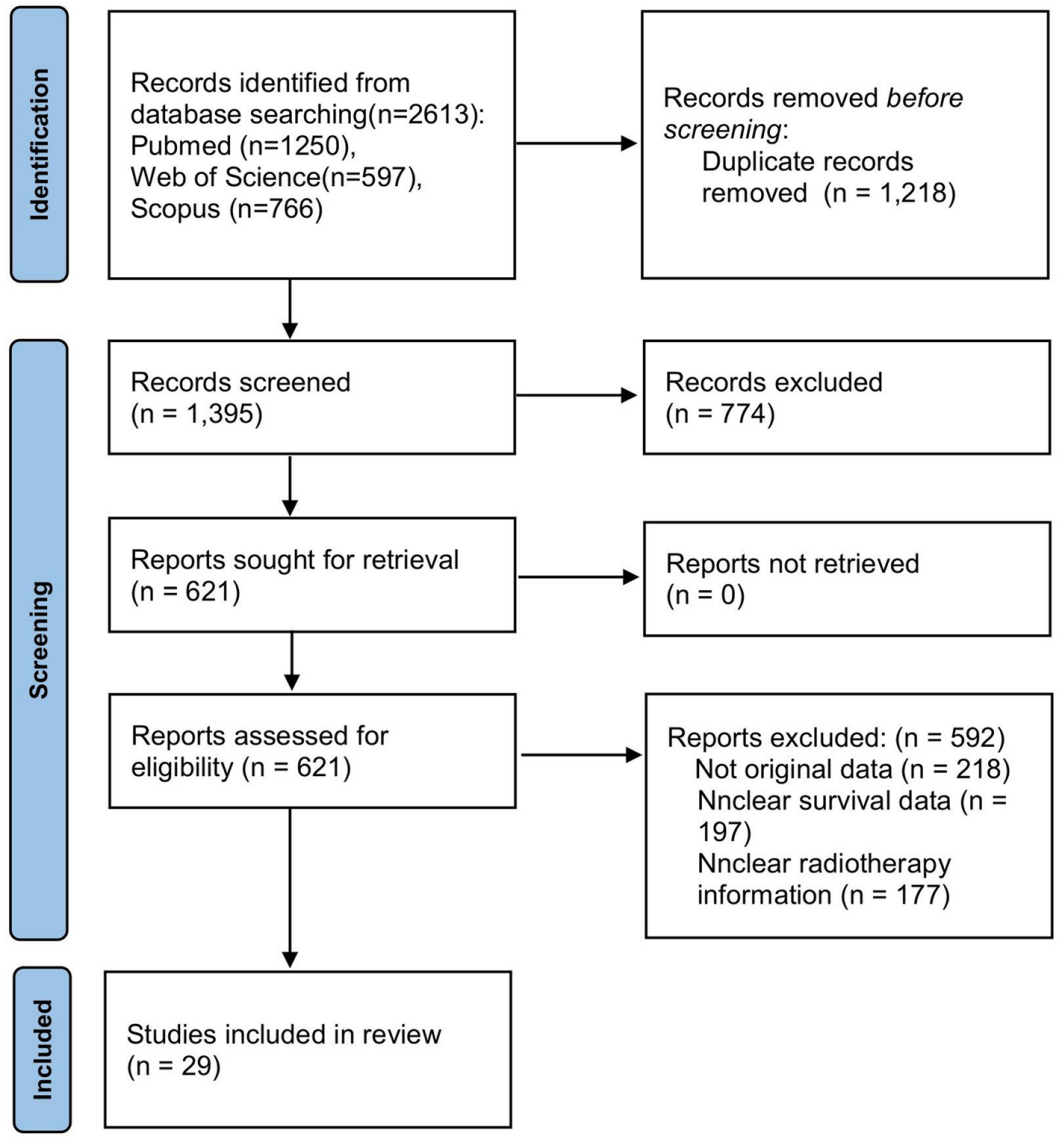
PRISMA flowchart of the process for study selection.

We prospectively registered the systematic review and meta-analysis with PROSPERO (CRD42022359669). This systematic review and meta-analysis was conducted in accordance with PRISMA (Preferred Reporting Items for Systematic Reviews and Meta-Analyses) guidelines^15^. The 2020 PRISMA flowchart and PRISMA checklist can be found in Fig. 1 and Table S1.

For the meta-analysis, a systematic literature search of the PubMed, Web of Science and Scopus databases (last search updated on September 2021) was conducted to identify studies that evaluated the association between the expression of lncRNAs and responses to radiotherapy and the prognosis of different tumors. The following search terms were used: “lnc RNA or long noncoding RNA” and “radioresistance or radiosensitivity or radiotherapy resistance or radiotherapy sensitivity or radioresistance or radiosensitivity” and “cancer or tumor or carcinoma”. In addition, a manual search for other relevant articles was performed using the reference lists of eligible studies.

### Selection criteria

Papers were defined as eligible studies if they met the following criteria: (1) studies of human clinical trials; (2) lncRNA involvement in radioresistance or radiosensitivity data in cancers; (3) quantitative measurement of lncRNA expression in tumor tissues; (4) the association between lncRNA expression and survival was estimated; and (5) sufficient data for the assessment of hazard ratios (HRs) and 95% confidence intervals (CIs).

The exclusion criteria were as follows: (1) only cell or animal experiments, case reports, editorials, letters, meta-analyses, or reviews; (2) articles where the required data could not be extracted from the original papers; (3) nonrelated studies to radiotherapy; (4) studies without a definite patient sample size; and (5) lack of sufficient data to calculate HR and 95% CI.

### Data extraction and quality assessment

The following data were extracted from all included studies: the first author’s name, year of study or publication, country, tumor type, involved lncRNAs, number and type of samples, patient gender, detection methods for lncRNA expression, cut-off level, outcomes, lncRNA expression and function, type of radiotherapy response, associated microRNAs and pathways. The prognostic endpoints included overall survival (OS), recurrence-free survival (RFS), disease-free survival (DFS) and progression-free survival (PFS). Additionally, the HR estimates were calculated from Kaplan–Meier survival curves using Engauge Digitizer V4.1 software according to the method of Tierney^16^ if these were not available (https://sourceforge.net/projects/digitizer/). Quality assessment of the primary studies was executed using the Newcastle–Ottawa Quality Assessment Scale^17^.

### Statistical analysis

A Cochrane’s Q test was implemented to test heterogeneity among studies. The p value of the Q test was < 0.1, which suggested the presence of heterogeneity, and the random effects model (DerSimonian–Laird method)^18^ was used to calculate pooled HRs. Otherwise, heterogeneity was absent, and the fixed effects model (Mantel–Haenszel method)^19^ was more appropriate and applied. In addition, the degree of heterogeneity was assessed by the I^2^ test. The value of I^2^ ranged from 0 to 100% and was generally considered no heterogeneity for I^2^ = 0, moderate heterogeneity for 25%, large heterogeneity to 50%, and extreme heterogeneity for 75%^20^. Furthermore, a funnel plot and Begg and Egger’s tests^21–23^ were utilized to investigate any possible publication bias. The funnel plot was visually symmetrical, and the P value of Begger’s or Egger’s test was greater than 0.05, which indicated that there was no statistically significant publication bias. Sensitivity analysis was performed by consecutive omission of individual studies to verify the consistency of outcomes. Statistical analyses were performed with STATA software version 14.0 (https://www.stata.com/) and Review Manager 5.3 software (http://www.cochrane.org/cochrane/download.htm).

## Results

### Study selection and characteristics of eligible studies

The primary search strategy yielded a total of 2613 publications from PubMed (n = 1250), Web of Science (n = 597) and Scopus (n = 766), 1395 of which were excluded due to duplication. According to the inclusion and exclusion criteria, 29 studies about the association between lncRNAs and radiosensitivity or radioresistance in various cancers were ultimately used for this meta-analysis, as shown in Fig. 1. The main characteristics of the included studies are summarized in Table 1. A total of 2602 patients between 2015 and 2021 were included. They all came from China. The malignant tumors in the study included head and neck squamous cell carcinoma (HNSCC)^24,25^, nasopharyngeal carcinoma (NPC)^26–32^, glioma^33,34^, laryngeal carcinoma (LC)^35^, esophageal cancer (EC)^36–38^, non-small-cell lung cancer (NSCLC)^39–42^, lung adenocarcinoma (LUAD)^43^, cardiac cancer (CaC)^44^, colorectal cancer (CRC)^13,45^, cervical cancer (CeC)^46,47^, breast cancer (BrC)^12,48,49^, and bladder cancer (BlC)^50^. The level of lncRNA expression was measured with quantitative reverse transcription polymerase chain reaction (qRT–PCR) and/or in situ hybridization (ISH) in the included studies. The cut-off score was the median value when using qRT–PCR. In ISH, a score of simultaneous immunohistochemistry (SI) < 6 was considered low expression (Table 1).

**Table 1:** Characteristics of studies included in this meta-analysis.

First author	Published year	Country	Tumour type	LncRNA	No. (radiosensitive/radioresistat) or (low/high)	Gender (male/female)	Sample	Detection	Cut off (%)	Outcomes
Gou C^24^	2020	China	HNSCC	BLACAT1	73 (36/37)	49/24	Tissue	qRT–PCR	Median	OS
Han Y^29^	2020	China	NPC	LINC00114	70	NR	Tissue	qRT–PCR	Median	OS
Lu Y^30^	2016	China	NPC	NEAT1	131 (46/85)	60/67	Tissue	ISH	SI < 6 SI ≥ 6	OS
Jin C^31^	2015	China	NPC	MALAT1	131 (65/66)	60/71	Tissue	ISH	SI < 6 SI ≥ 6	OS
Han Y^26^	2020	China	NPC	PVT1	29 (14/15)	16/13	Tissue	qRT–PCR	Median	OS
Zhong Q^32^	2021	China	NPC	MINCR	49 (25/24)	NR	Tissue	qRT–PCR	Median	OS
Zheng J^33^	2020	China	Glioma	Linc-RA1	120 (57/63)	71/49	Tissue	qRT–PCR	Median	OS, PFS
Han P^25^	2018	China	HNSCC	LINC00473	78 (38/40)	NR	Tissue	qRT–PCR	Median	OS
Guo Z^28^	2021	China	NPC	LINC00312	81 (41/40)	NR	Tissue	qRT–PCR	Median	OS
Tang T^34^	2020	China	Glioma	TPTEP1	177 (96/81)	111/66	Tissue	ISH	Median	OS, PFS
Yang T^35^	2018	China	LC	NKILA	65 (32/33)	49/16	Tissue	qRT–PCR	Median	OS
He Y^27^	2018	China	NPC	PVT1	76 (25/51)	NR	Tissue	qRT–PCR	Median	RFS
Liu AM^39^	2019	China	NSCLC	FAM201A	69 (34/35)	47/22	Tissue	qRT–PCR	Median	OS
Han F^43^	2020	China	LUAD	LINC00857	87	56/31	Tissue	qRT–PCR	Median	OS
Wu D^41^	2017	China	NSCLC	PVT1	31 (16/15)	22/9	Tissue	qRT–PCR	Median	OS
Qin P^40^	2020	China	NSCLC	LINC00473	72 (40/32)	NR	Tissue	qRT–PCR	Median	OS
Zhang J^42^	2018	China	NSCLC	CYTOR	64 (32/32)	38/26	Tissue	qRT–PCR	Median	OS
Chen W^36^	2019	China	ESCC	LINC00473	96 (49/47)	NR	Tissue	qRT–PCR	Median	OS
Wang M^61^	2019	China	EC	CCAT2	60 (26/34)	38/22	Tissue	qRT–PCR	Median	OS
Li Z^38^	2020	China	EC	Rpph1	83 (42/41)	48/35	Tissue	qRT–PCR	Median	OS
Liu Y^13^	2020	China	CRC	HOTAIR	71 (35/36)	NR	Tissue	qRT–PCR, ISH	Median	OS
Liang H^45^	2021	China	CRC	LINC00958	63	NR	Tissue	qRT–PCR	Median	OS, DFS
Jia J^44^	2019	China	CaC	H19	284 (93/191)	176/108	Tissue	qRT–PCR	Median	OS
Liu L^48^	2019	China	BrC	LINC00511	98 (49/49)	0/98	Tissue	qRT–PCR	Median	OS
Wang B^12^	2019	China	BrC	LINC02582	136 (65/71)	NR	Tissue	qRT–PCR	Median	RFS
Bi Z^49^	2020	China	TBNC	AFAP1-AS1	125 (61/64)	0/125	Tissue	ISH	Median	OS, DFS
Tan J^50^	2015	China	BlC	TUG1	54	36/18	Tissue	qRT–PCR	Median	OS
Han D^47^	2018	China	CeC	NEAT1	72 (36/36)	37/35	Tissue	qRT–PCR	Median	OS
Zhao H^46^	2019	China	CeC	LINC00958	57 (31/26)	0/57	Tissue	qRT–PCR	Median	OS

*NR* not reported, *HNSCC* head and neck squamous cell carcinoma *NPC* nasopharyngeal carcinoma, *ESCC* esophageal squamous cell carcinoma, *EC* esophageal cancer, *LC* laryngeal carcinoma, *NSCLC* non-small cell lung cancer, *LUAD* lung adenocarcinoma, *TBNC* triple-negative breast cancer, *CaC* cardiac cancer, *CRC* colorectal cancer, *CeC* cervical cancer, *BrC* breast cancer, *BlC* bladder cancer, *qRT–PCR* quantitative reverse transcription polymerase chain reaction, *ISH* in situ hybridization, *SI* simultaneous immunohistochemistry, *OS* overall survival, *RFS* recurrence-free survival, *DFS* disease-free survival, *PFS* progression-free survival.

### Association between lncRNAs and radiotherapy response in vitro

**Figure 2:**
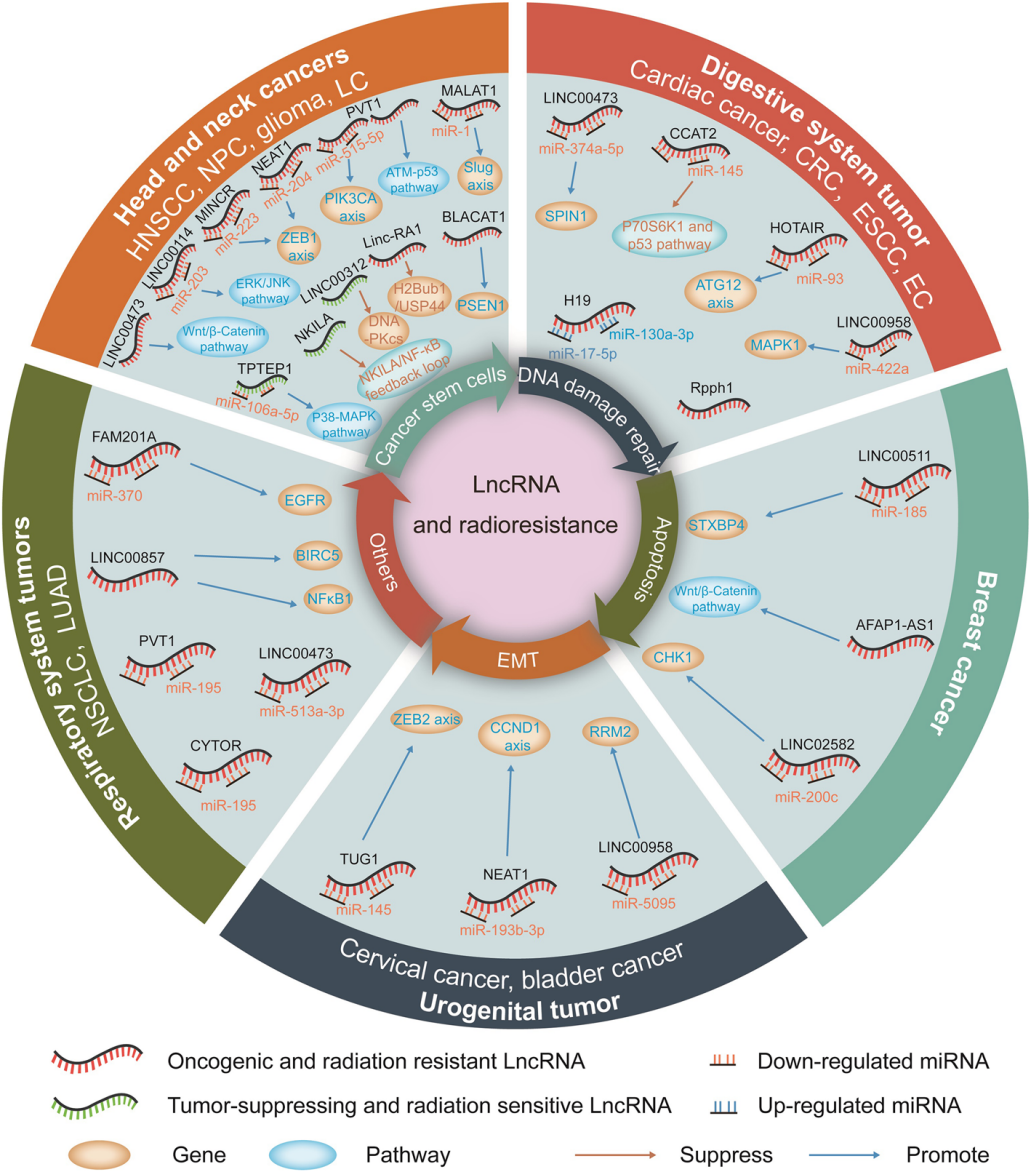
The mechanisms and targets of lncRNAs involved in regulating radiosensitivity and radioresistance in various types of malignant tumors.

There were 23 lncRNAs in 11 cancer types included in our systematic review and meta-analysis. Of them, 13 lncRNAs were downregulated by small-interfering RNA (siRNA) transfection, and 14 were upregulated by lentiviral vector infection. Furthermore, 16 lncRNAs were observed to be related to radiosensitivity, while 11 were found to be associated with radioresistance. Reduced expression of BLACAT1 in HNSCC; MALAT1, NEAT1 and PVT1 in NPC; FAM201A, PVT1 and LINC00857 in NSCLC; LINC00473, CCAT2and Rpph1 in EC; HOTAIR and LINC00958 in CRC; AFAP1-AS1 and LINC00511 in breast cancer were correlated with radiosensitivity. Increased expression of LINC00473 in HNSCC; LINC00114, MINCR and PVT1 in NPC; Linc-RA1 in glioma; LINC00473and CYTOR in NSCLC; NEAT1 and LINC00958 in cervical cancer; H19 in cardiac cancer; LINC02582 in breast cancer; and TUG1 in bladder cancer were associated with radioresistance. However, some elevated expression of LINC00312 in NPC, TPTEP1 in glioma, and NKILA in laryngeal carcinoma was found to be related to radiosensitivity. Four lncRNAs appeared in more than one type of cancer and were differentially regulated, including downregulation of LINC00473 in ESCC radiosensitivity and upregulation in HNSCC and NSCLC radioresistance, downregulation of NEAT1 in NPC radiosensitivity and upregulation in cervical cancer radioresistance, downregulation of LINC00958 in colorectal cancer radiosensitivity and upregulation in cervical cancer radioresistance, downregulation of PVT1 in NPC and NSCLC radiosensitivity and upregulation in NPC radioresistance. Additionally, 17 miRNAs and 20 pathways or genes were targeted by different lncRNAs, which contributed to DNA damage repair, apoptosis, CSC regulation, EMT, etc., causing cancer cell radioresistance or sensitivity (Table 2 and Fig. 2).

**Table 2:** LncRNA-microRNA pathways related to radiosensitivity and radioresistance.

Tumour type	LncRNA	Expression	Function	Radiotherapy response	MicroRNA	Target or pathway
HNSCC^15^	BLACAT1	Downregulated	DNA damage repair, apoptosis, viability cycle arrest	Radiosensitivity	NR	PSEN1
NPC^17^	PVT1	Downregulated	Proliferation, apoptosis	Radiosensitivity	miR-515-5p	PIK3CA axis
NPC^21^	NEAT1	Downregulated	EMT	Radiosensitivity	miR-204	ZEB1 axis
NPC^22^	MALAT1	Downregulated	CSC	Radiosensitivity	miR-1	Slug axis
NSCLC^30^	FAM201A	Downregulated	Proliferation, apoptosis	Radiosensitivity	miR-370	EGFR
NSCLC^32^	PVT1	Downregulated	Proliferation, apoptosis	Radiosensitivity	miR-195	NR
LUAD^34^	LINC00857	Downregulated	Proliferation, apoptosis	Radiosensitivity	NR	BIRC5/NFκB1
ESCC^27^	LINC00473	Downregulated	Proliferation	Radiosensitivity	miR-374a-5p	SPIN1
EC^28^	CCAT2	Downregulated	Apoptosis	Radiosensitivity	miR-145	P70S6K1 and p53 pathway
EC^29^	Rpph1	Downregulated	Apoptosis, migration, EMT, proliferation	Radiosensitivity	NR	NR
CRC^13^	HOTAIR	Downregulated	Viability, apoptosis, autophagy	Radiosensitivity	miR-93	ATG12 axis
CRC^36^	LINC00958	Downregulated	Proliferation, apoptosis	Radiosensitivity	miR-422a	MAPK1
TBNC^40^	AFAP1-AS1	Downregulated	Proliferation, migration, invasion	Radiosensitivity	NR	Wnt/β-Catenin pathway
BrC^39^	LINC00511	Downregulated	Proliferation, apoptosis	Radiosensitivity	miR-185	STXBP4
NPC^20^	LINC00114	Upregulated	Proliferation, migration	Radioresistance	miR-203	ERK/JNK pathway
NPC^23^	MINCR	Upregulated	Viability, apoptosis	Radioresistance	miR-223	ZEB1 axis
NPC^18^	PVT1	Upregulated	DNA damage, apoptosis, proliferation	Radioresistance	NR	ATM-p53 pathway
HNSCC^16^	LINC00473	Upregulated	Proliferation, apoptosis	Radioresistance	NR	Wnt/β-catenin pathway
Glioma^24^	Linc-RA1	Upregulated	DNA damage, autophagy	Radioresistance	NR	H2Bub1/USP44
NSCLC^31^	LINC00473	Upregulated	Proliferation	Radioresistance	miR-513a-3p	NR
NSCLC^33^	CYTOR	Upregulated	Viability, apoptosis	Radioresistance	miR-195	NR
CaC^35^	H19	Upregulated	Proliferation, viability, apoptosis	Radioresistance	miR-130a-3p/miR-17-5p	NR
BlC^46^	TUG1	Upregulated	EMT	Radioresistance	miR-145	ZEB2 axis
CeC^38^	NEAT1	Upregulated	Proliferation, cycle arrest, apoptosis	Radioresistance	miR-193b-3p	CCND1 axis
CeC^37^	LINC00958	Upregulated	Proliferation, apoptosis	Radioresistance	miR-5095	RRM2
BaC^12^	LINC02582	Upregulated	–	Radioresistance	miR-200c	CHK1
NPC^19^	LINC00312	Upregulated	DNA damage repair, cell cycle	Radiosensitivity	NR	DNA-PKcs
Glioma^25^	TPTEP1	Upregulated	CSC	Radiosensitivity	miR-106a-5p	P38/MAPK pathway
LC^26^	NKILA	Upregulated	Viability, migration, apoptosis	Radiosensitivity	NR	NKILA/NF-κB feedback loop

*NR* not reported, *HNSCC* head and neck squamous cell carcinoma, *NPC* nasopharyngeal carcinoma, *ESCC* esophageal squamous cell carcinoma, *EC* esophageal cancer, *LC* laryngeal carcinoma, *NSCLC* non-small cell lung cancer, *LUAD* lung adenocarcinoma, *TBNC* triple-negative breast cancer, *CaC* cardiac cancer; *CRC* colorectal cancer, *CeC* cervical cancer, *BrC* breast cancer, *BlC* bladder cancer, *CSC* cancer stem cell, *EMT* epithelial to mesenchymal transition.

### Association between OS and lncRNA expression with regard to radiotherapy response

**Figure 3:**
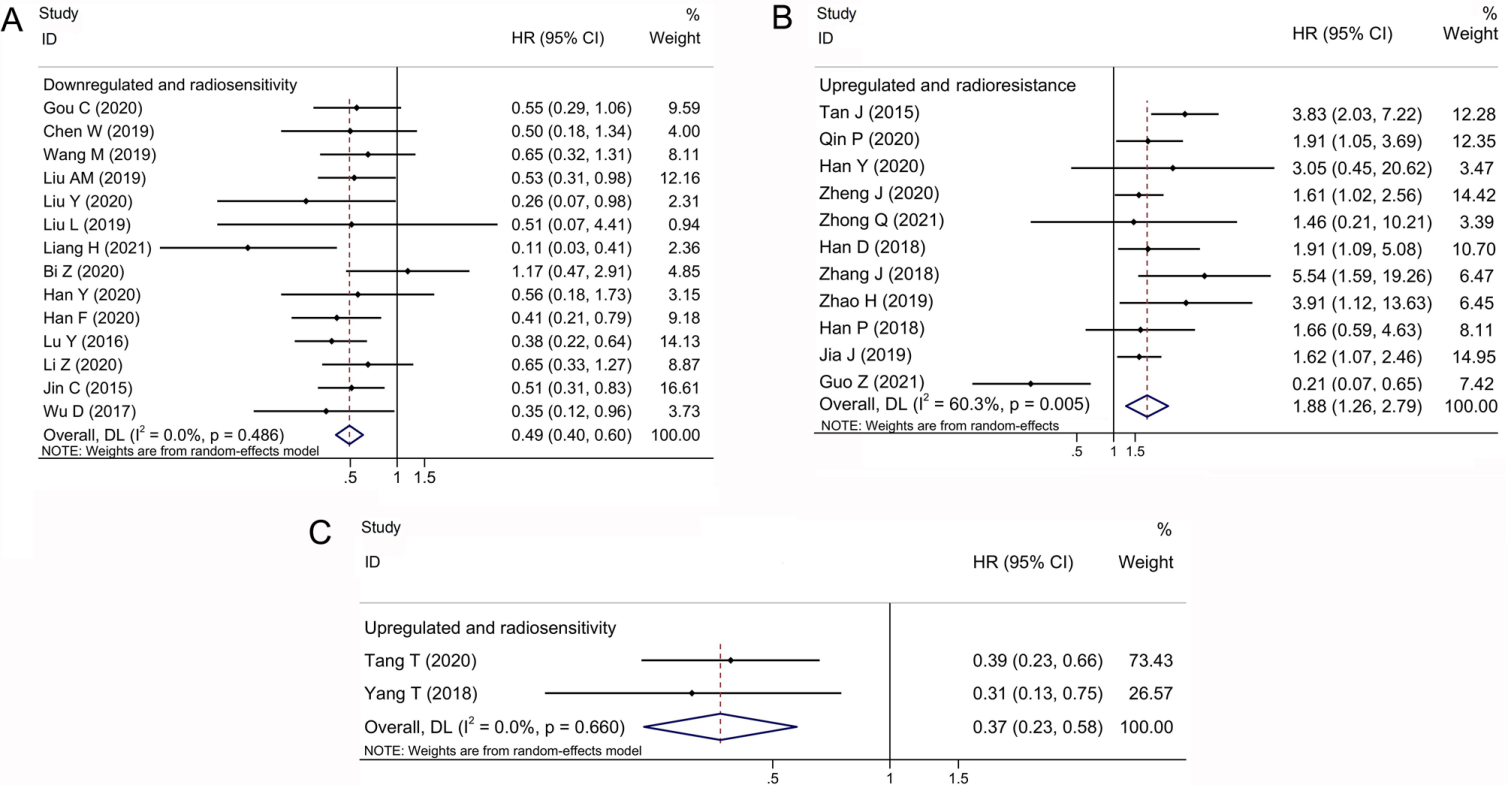
Forest plot for the association between lncRNA expression levels and OS. *OS* overall survival, *HR* hazard ratio, *CI* confidence interval.

A total of 27 studies were included for OS analysis. Under the random-effects model, the individual overall HR (95% CI) of downregulated radiotherapyresistant lncRNAs was 0.49 (0.40–0.60), with extreme heterogeneity (I^2^ = 0.0%; P_h_ = 0.486) (Fig. 3A). The individual overall HR (95% CI) of upregulated radiotherapy-resistant lncRNAs was 1.88 (1.26–2.79) (Fig. 3B). The individual overall HR (95% CI) of upregulated radiotherapy-sensitive lncRNAs was 0.37 (0.23–0.58) from few studies (Fig. 3C). Our results showed that downregulated radiotherapy-resistant lncRNAs were associated with better OS in cancer patients treated with radiotherapy. Similar patterns were found for DFS (random-effects model: pooled HR 0.28; 95% CI 0.10–0.75), although there were few qualified articles included in the analysis. However, no significant difference was found in the RFS and PFS analyses (Fig. S1).

### Subgroup analysis of the association between OS and lncRNA expression with regard to radiotherapy response

**Figure 4:**
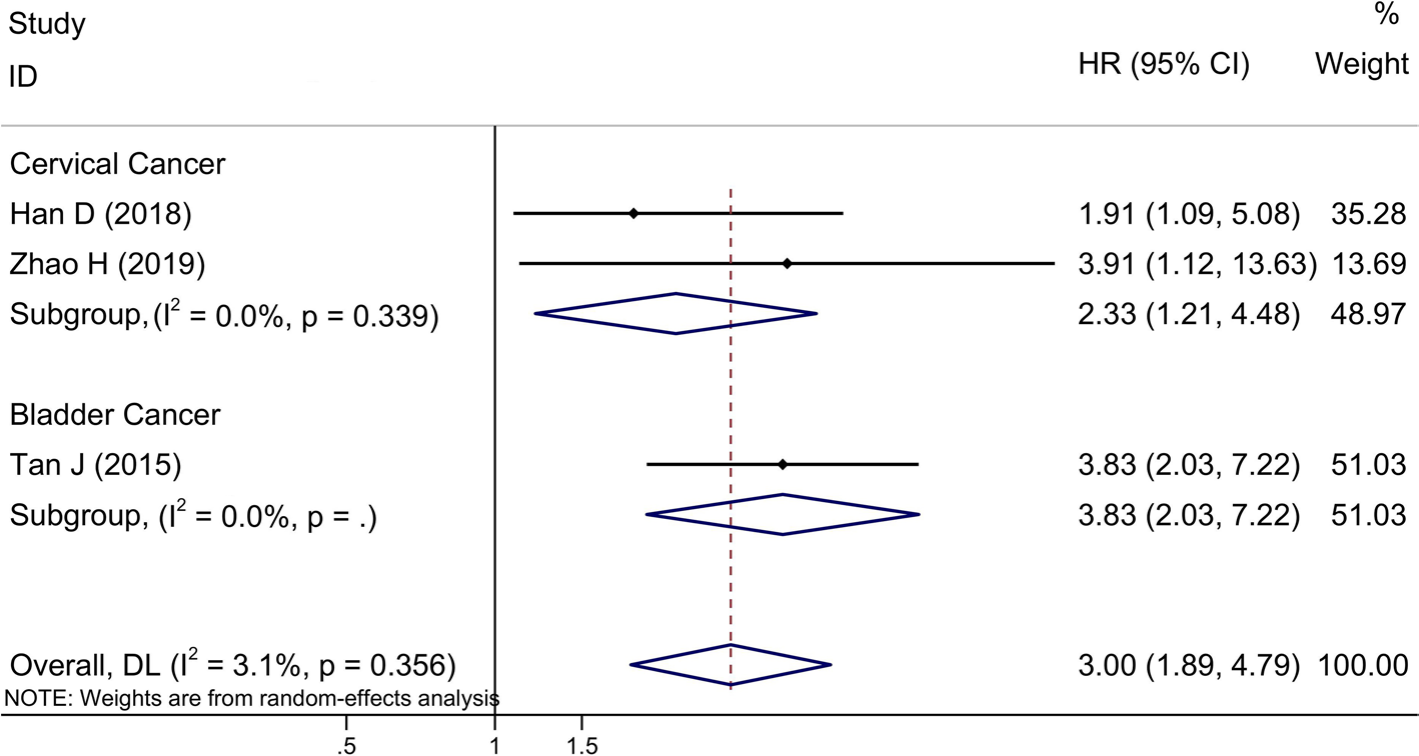
Forest plot for the association between lncRNA expression levels and OS according to tumor type, including cervical cancer and bladder cancer. *OS* overall survival, *HR* hazard ratio, *CI* confidence interval.

Subgroup analyses were performed based on the type of cancer. Head and neck cancers included HNSCC, NPC, glioma and LC; respiratory system tumors included NSCLC and LUAD; and digestive tract system tumors included cardiac cancer, CRC and EC. Under the random-effects model, the HR and 95% CI of upregulated radiotherapy-resistant lncRNAs were 2.33 (1.21–4.48) for cervical cancer studies, and 3.83 (2.03–7.22) for bladder cancer studies (Fig. 4). Our results showed that upregulated radiotherapy-resistant lncRNAs were associated with worse OS in these cancer patients treated with radiotherapy.

However, for head and neck cancer studies, the individual HR (95% CI) of downregulated radiotherapy-resistant lncRNAs was 0.47 (0.35–0.64), the individual HR (95% CI) of upregulated radiotherapy-resistant lncRNAs was 1.66 (1.11–2.48) and the individual HR (95% CI) of upregulated radiotherapy-sensitive lncRNAs was 0.34 (0.22–0.52), respectively (Fig.S2). For breast cancer studies, the individual HR (95% CI) of downregulated radiotherapy-resistant lncRNAs was 1.02 (0.44–2.36). The results implied that there was no statistically significant in the association of radiotherapy-related lncRNAs and survival in breast cancer (Fig. S2).

Furthermore, in the respiratory system and digestive tract system subgroups, the individual HRs (95% CIs) of downregulated radiotherapy-resistant lncRNAs were 0.45 (0.30–0.68) and 0.43 (0.25–0.76), respectively. Correspondingly, the HRs of upregulated radiotherapy-resistant lncRNAs were 2.82 (1.03–7.72) and 1.62 (1.07–2.46), respectively. The results also indicated that downregulated radiotherapy-resistant lncRNA expression was associated with radiosensitivity and could be a better prognostic marker for those patients (Fig. S2).

### Publication bias

**Figure 5:**
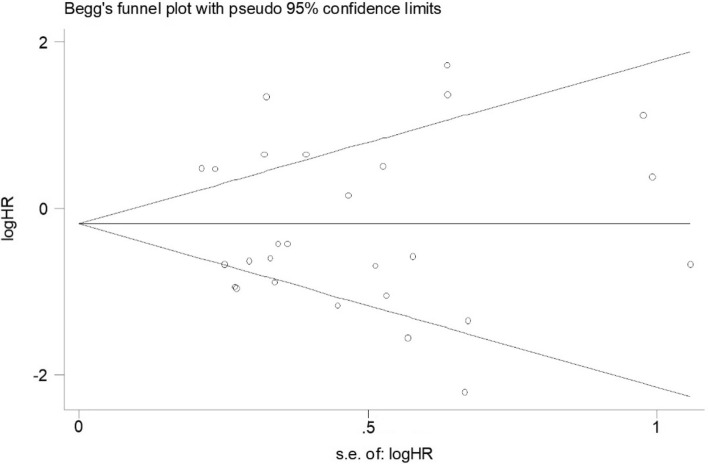
Begg’s funnel plots of publication bias for OS in all eligible studies. Each circle represents a separate study. *OS* overall survival.

Begg’s funnel plot and Egger’s test were performed to detect publication bias. There was no obvious asymmetry in Begg’s funnel plots of OS (Fig. 5, p = 0.967). The p values of Egger’s tests were all greater than 0.05, indicating no potential publication bias in our study.

### Sensitivity analysis

To assess the stability of the pooled results in the current meta-analysis, we conducted a sensitivity analysis by gradually removing each single study from the merged analysis. The results indicated that the overall pooled estimates could not be affected by a single study (Fig. S3).

## Discussion

Radiotherapy is considered a highly effective cancer treatment. Radiotherapy resistance is still the main cause of treatment failure, leading to tumor recurrence and metastasis. Tumor and microenvironment heterogeneity is considered to be responsible for the differing sensitivities of tumor cells to cancer treatment, including radiotherapy, which explains why tumor subpopulations are not equally affected by this treatment^51,52^. Therefore, it is crucial to explore the targets and mechanisms of radiotherapy resistance, providing some potential therapeutic points for improving cancer radiotherapy.

Various studies have confirmed that deregulated lncRNAs contribute to radiotherapy resistance by regulating abnormal DDR, apoptosis, CSCs and EMT in many cancers, such as NPC, breast cancer, NSCLC, ESCC, cervical cancer and colorectal cancer^12,29,42,45,46,53,54^. In the current study, we performed the first comprehensive systematic review and meta-analysis aimed to evaluate the effect of lncRNA expression on radiotherapy response and patient prognosis in various human cancers.

Our meta-analysis showed that lncRNAs with lower expression were significantly associated with a better radiotherapy response and OS, while lncRNAs with higher expression were related to radiotherapy resistance. In the subgroup analysis, based on cancer type, lncRNAs were determined to be a potential predictor for prognosis in various cancers, includingseveral neoplasms of the genitourinary system (e.g., cervical and bladder cancers)^46,47,55^. Previous studies have shown that urothelial cells in bladder cancer have high radioresistance. LncRNAs may act as potential regulators of radioresistance in these cancers, and they may represent promising therapeutic targets by interfering with their expression^55^. Most head and neck cancers are radiation-sensitive squamous carcinomas. However, the impact of radiotherapy-related lncRNAs on the prognosis of patients with head and neck cancers is diverse and inconsistent. In addition, we could not find a significant role of lncRNAs in the radiotherapy response for breast cancer due to insufficient studies reporting clinical outcomes^12,48,49^. More research needs to be done to clarify the effect of lncRNAs on radiotherapy in these cancers.

Furthermore, lncRNAs mediate the tumor response to radiotherapy by acting on a wide range of target genes and pathways. Numerous studies have confirmed that lncRNAs may serve as endogenous sponges to regulate the expression and function of miRNAs and subsequently radiotherapy regulatory pathways^10,37,42^. We found that more than one lncRNA/miRNA interaction has been investigated in distinctive cancer cell lines and tissues (e.g., NPC, NSCLC, CRC, cervical cancer and breast cancer). NPC, where radiotherapy is the main and essential treatment, has been the most studied to explore the effect of lncRNAs on radiotherapy^26–32^. In NPC, PVT1 regulates DNA repair and cell apoptosis to induce radioresistance via the miR-515-5p/PIK3CA axis and modulates the AKT pathway by interacting with miR-515-5p^26^. MALAT1 modulates CSC activity and radioresistance by inactivating miR-1^31^. NEAT1 modulates EMT and radioresistance through the miR-204/ZEB1 axis^30^. Thus, lncRNAs could act as promising theragnostic biomarkers for predicting radioresistance and radiosensitivity. In addition, a previous meta-analysis indicated that miRNAs contribute to the cellular response to irradiation by affecting radiation-related signaling pathways, such as the PI3K/AKT/mTOR pathway^56^. It seems that miRNAs accompanied by lncRNAs act as theragnostic biomarkers to predict and monitor the clinical response to radiotherapy. In addition, specific lncRNAs can also appear in different types of tumors. For example, LINC00473 facilitated radioresistance in ESCC, HNSCC and NSCLC by modulating the miR‐374a‐5p/SPIN1 axis, activating the Wnt/β-catenin pathway and sponging miR-513a-3p, respectively^25,36,40^. Numerous studies have proven that LINC00473, a novel lncRNA, is upregulated and acts as an oncogene in most human cancers, leading to tumor progression^57,58^. Furthermore, the overexpression of LINC00473 is strongly associated with poor survival^59,60^. Our study showed that LINC00473 regulated the sensitivity of radiotherapy for several different neoplasms^25,36,40^. In addition, the MAPK pathway and p53 pathway were found to be the main mechanisms involved in the regulation of the radiotherapy response mediated by lncRNAs.

There were several limitations in our meta-analysis. Heterogeneity was observed to be high within the forest plots of the total lncRNAs and some subgroups, suggesting that HRs vary across studies. The indications of radiation therapy in different cancers and sometimes in different stages of the same cancer type are different. It can be with curative intent, palliative, adjuvant or neoadjuvant, etc. which should contribute to the heterogeneity among the studies. Thus, the random-effects model was applied in these analyses. This could be explained by the number of patients, various types of tumors, variable cut-off points and, most importantly, the upregulation and downregulation of lncRNA expression integrated for calculation. Fortunately, according to the sensitivity analyses, we could suggest that, despite heterogeneity, the pooled HR can be considered quite reliable and representative. Although we performed a systematic and extensive literature search, the studies included only Asian patients and caused singleness of the population. Thus, there were not enough eligible studies for all types of cancers involving radiotherapy (e.g., lack of soft tissue tumors, lymphoma, etc.). Additionally, only nearly 14% of the included papers had direct hazard values that could be applied for the current meta-analysis, leading to bias and decreased accuracy of the results. The HR for OS in our study was retrieved by KM curve; thus, this is a univariable analysis. However, HR should be extracted by multivariable analysis to avoid selection bias and remove confounders as much as possible. These results suggest that further associated studies are needed to precisely determine the effect of lncRNA expression on radiotherapy response and prognosis.

## Conclusion

In conclusion, this comprehensive systematic review and meta-analysis showed the role of lncRNA expression on radiotherapy response and survival in various malignancies. Furthermore, we provided lists of potential radiation-related signaling pathways controlled by lncRNA/miRNA interactions that might help to identify new therapeutic targets to counter radioresistance.

## Supplementary Information

Supplementary Information.

## Data Availability

All data generated or analyzed during this study are included in this published article and the Supplementary Information files.

## Abbreviations

BrC: Breast cancer
BlC: Bladder cancer
CSC: Cancer stem cell
CHK1: Checkpoint kinase 1
CaC: Cardiac cancer
CRC: Colorectal cancer
CeC: Cervical cancer
DFS: Disease-free survival
DSB: Double-strand break
DDR: DNA damage response
EMT: Epithelial-mesenchymal transition
ESCC: Esophageal squamous cell carcinoma
EC: Esophageal cancer
HR: Hazard ratio
HNSCC: Head and neck squamous cell carcinoma
ISH: In situ hybridization
lncRNA: Long noncoding RNA
LC: Laryngeal carcinoma
LUAD: Lung adenocarcinoma
miRNA: MicroRNA
NSCLC: Non-small-cell lung cancer
NPC: Nasopharyngeal carcinoma
OS: Overall survival
PFS: Progression-free survival
PRISMA: Preferred Reporting Items for Systematic Reviews and Meta-Analyses
qRT–PCR: Quantitative reverse transcription polymerase chain reaction
RFS: Recurrence-free survival
ROS: Reactive oxygen species
SSB: Single-strand break
SI: Simultaneous immunohistochemistry
siRNA: Small-interfering RNA
USP7: Ubiquitin specific peptidase 7
